# Acute visual impairment as a main presenting symptom of non-convulsive status epilepticus: a case report

**DOI:** 10.1186/s12883-020-1630-x

**Published:** 2020-02-11

**Authors:** Yi Yang, Shunyuan Zhang, Jinfeng Duan, Xianwen Zhang, Yufeng Tang

**Affiliations:** 1 Department of Neurology, Mianyang Central Hospital, Changjia Alley 12#, Fucheng district, Mianyang City, 621000 Sichuan China; 2 Department of Radiology, Mianyang Central Hospital, Mianyang City, China

**Keywords:** Acute visual impairment, NCSE, Hemianopsia and visual neglect, TPMA

## Abstract

**Background:**

Nonconvulsive status epilepticus (NCSE) is a state of ongoing seizure activity without convulsions. The heterogeneous and subtle clinical features of NCSE make diagnosis and treatment challenging. Here, we report a patient with NCSE who showed a main presenting symptom of acute visual impairment, which is a rare and atypical clinical symptom of NCSE.

**Case presentation:**

A 62-year-old man was admitted to the neurology department after complaining of an inability to see in the right eye for 2 days and progressive headache. He had a history of poststroke epilepsy and vascular dementia. Physical examination revealed right visual field hemianopia, visual neglect and cognitive impairment. T2 and diffusion-weighted magnetic resonance imaging showed high signal intensity in the left temporal, parietal and occipital lobes. Electroencephalography monitoring was performed, which found continuous sharp wave discharges, especially in the regions of the left temporal, parietal and occipital lobes. These findings were most consistent with the diagnosis of NCSE. Thus, a treatment of intravenous pumping of diazepam and an oral antiepileptic drug was added immediately. After that, the visual loss in the patient recovered quickly, and electroencephalography did not find epileptiform waves. On day 11, a follow-up MRI was performed, which showed that the abnormal signals of the left temporal, parietal and occipital lobes were markedly attenuated, and the patient returned to his premorbid state with a modified Rankin Scale score of 3.

**Conclusions:**

Acute visual impairment can be seen in NCSE, and it can be reversed by administering effective antiepileptic treatment. Meanwhile, transient peri-ictal MRI abnormalities can be observed in NCSE.

## Background

Nonconvulsive status epilepticus (NCSE) represents a major problem in neurological critical care patients. The variable and subtle clinical symptoms of NCSE, such as staring, repetitive blinking, chewing, swallowing, automatism, altered mental status, subtle facial or limb twitches, speech disturbance and extrapyramidal signs, make diagnosis and treatment challenging [1]. Continuous seizure activity can have long-term consequences (after time point t2), including neuronal injury, neuronal death, and alteration of neuronal networks [2]. A heightened awareness of the disease and continuous electroencephalography (EEG) monitoring have been associated with an early diagnosis of NCSE [3]. Acute visual loss had previously been reported in a patient with occipital lobe epilepsy as the initial symptom of mitochondrial encephalomyopathy, lactic acidosis and stroke-like episodes (MELAS) [4]. However, it is a rare and atypical clinical symptom of NCSE. In addition, during the acute ictal and postictal phases of seizures, acute MR imaging features of seizures may occur, which are named transient peri-ictal MRI abnormalities (TPMA) [5]. Here, we report a patient with NCSE who presented with acute visual impairment and TPMA.

### Case presentation

**Fig. 1:**
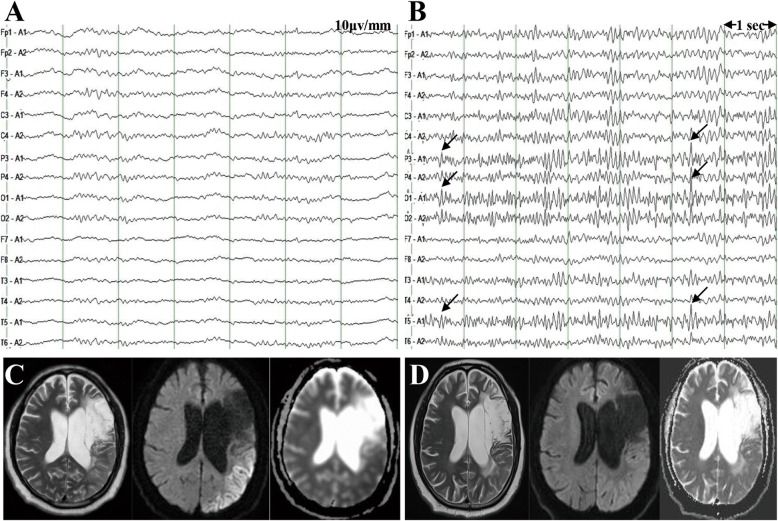
EEG recordings showed that the interictal EEG background was normal (**a**), and ictal EEG showed continuous sharp wave discharges mainly in the regions of the left temporal, parietal and occipital lobe (**b**). A follow-up MRI (**d**) showed that the lesions of the left parietooccipital lobe were markedly attenuated compared to the MRI performed on admission (11 days ago) (**c**)

A 62-year-old man was admitted to the neurology department complaining of an inability to see in the right eye for 2 days along with acute headache. He had a history of atrial fibrillation in rheumatic heart disease and suffered an ischaemic stroke in the left frontotemporal parietal lobe 4 years ago. The right limb of the patient was left with sequelae of mild paralysis, and he gradually developed mild vascular dementia; however, he could communicate with family members normally in daily life. He suffered symptomatic epilepsy due to ischaemic stroke 2 years ago, and effective antiepileptic drugs (500 mg levetiracetam bid and 500 mg valproic acid bid) were prescribed. At the first medical evaluation, the arterial pressure was 170/100 mmHg, and arterial blood lactic acid was 3.3 mmol/L. Liver function, renal function, electrolytes, erythrocyte sedimentation rate, procalcitonin, cerebrospinal fluid examination, syphilis antibody and HIV testing were normal. Neurological examination revealed a cognitive decline wherein the patient could not accurately answer questions or simply reply with short sentences or words. Ophthalmological examination found that when the patient’s eyes were unshielded, he needed to walk with help to distinguish orientations. Neither eye of the patient could read a visual chart clearly, but he could grasp a pen when shaken in front of both eyes, suggesting macular avoidance or partial visual field avoidance. The patient suffered sudden visual impairment, acute headache and progressive dementia, which suggested that the lesion may be located in the cerebral cortex. An MRI was then performed, which showed marked diffuse cortical hyperintensities in the left temporoparietal and occipital lobes on T2 and diffusion-weighted images. The apparent diffusion coefficient map demonstrated decreased signal intensity in the area of interest compared with the control region. Thus, the lesion was identified, while the differential diagnosis was relatively difficult. The patient had rheumatic heart disease and atrial fibrillation, which were risk factors for arterial cerebral infarction. However, CT angiography did not find corresponding vascular occlusion, and the lesion was not consistent with the vascular distribution. Thus, arterial cerebral infarction was excluded. The patient did not have drug or poison contact. Liver function, kidney function and plasma glucose were normal. Metabolism or toxic encephalopathy was less possible. Reversible posterior leukoencephalopathy syndrome (RPLS) was another possible diagnosis, as it could present with similar symptoms and the elevated blood pressure obtained at admission. However, the lesion was located on one side of the cortex, and the focus was a cytotoxic brain oedema. RPLS was less likely because its focus is often vasogenic oedema and symmetric. The lesion was located in the cortex of the left temporoparietal occipital lobes, and the lactate level was increased at admission. MELAS could not be completely ruled out. However, the patient did not have exercise intolerance in the past and had a rather older age. We found that the lactic acid level was normal. These clinical data did not suggest a diagnosis of MELAS. One day after hospitalization, the patient suffered limb twitches of the right lower limb lasting for approximately 1 min., and his wife told us that the patient was more taciturn than he had been in the past week, which she ignored at the beginning. Consequently, continuous EEG monitoring was performed, which found continuous sharp wave discharges, especially in the regions of the left temporal, parietal and occipital lobes. The patient had a history of epilepsy, acute headache, and visual and cognitive impairment. MRI found cytotoxic brain oedema in the left temporoparietal and occipital lobes, and EEG found sharp waves discharges mainly in the regions of the left central, parietal and occipital lobes. Taken together, these findings were most consistent with NCSE. Continuous intravenous administration of diazepam and oral addition of the antiepileptic drug topiramate (50 mg bid) were prescribed. After that, the patient recovered quickly, and follow-up EEG monitoring was normal. A follow-up MRI was performed 11 days later, which showed that the abnormal signal of the left parietooccipital occipital lobes was markedly attenuated (Fig. 1). We concluded that the abnormal and reversible imaging on the MRI was TPMA.

## Discussion and conclusions

Here, we report a patient with NCSE who presented with acute visual impairment as a main presenting symptom. For patients with an inability to see in the right eye on admission, the lesion may be located in front of the optic chiasma. However, the symptoms of acute headache and cognitive impairment could not be fully explained. MRI identified an abnormal signal in the left temporoparietal occipital lobes. It was presumed that the blindness of the right eye described by the patient was a misrepresentation of the true symptom, which may be caused by right visual field neglect combined with hemianopia. Visuospatial bias is associated with an inability to detect or respond to stimuli presented to the contralesional visual field. A study showed that unilateral spatial neglect could be a unique clinical expression of non-convulsive status epilepticus [6]. Anatomical lesion correlates of visual neglect are the temporoparietal junction (TPJ), including the inferior parietal lobule and superior temporal gyrus (STG), as well as the premotor cortex, basal ganglia and thalamus [7]. Visuospatial attention deficits are commonly seen as right-hemispheric disorders but have also been described after left-hemispheric damage, which is thought to be less frequent, less severe, and shorter lived than right-hemispheric lesions [8]. When visual field neglect is combined with hemianopia, mimicked monocular blindness may occur.

For patients with epilepsy, it is vital to collect the recent medical history, especially that related to epileptic seizures. Our patient had slow reactions a week prior, while his wife did not pay enough attention to the subtle clinical symptoms of epileptic seizure. However, the mild vascular dementia in the patient made his wife ignore his progressive cognitive impairment. Blinking, chewing, swallowing, subtle facial or limb twitches, head and/or eye deviation and automatism disturbances are classical features of NCSE, but coma, prolonged apnea, cardiac arrest, dementia and higher brain dysfunction may also be observed [1, 9]. In addition to progressive dementia, acute visual impairment could be another clinical symptom of NCSE if the occipital cortex is involved. Cortical blindness owing to occipital lobe epilepsy is relatively rare [10, 11]. For patients with atypical symptoms of NCSE, it is vital that the diagnosis of NCSE meet the EEG criteria [12]. Thus, for patients with acute visual impairment, continuous epileptic seizures should be considered an ineligible cause, especially when combined with other symptoms of brain damage.

Except for continuous EEG monitoring for NCSE, MRI may be a very useful neuroimaging technique to detect changes related to sustained epileptic activity. The diffusion-weighted magnetic resonance imaging and apparent diffusion coefficient (ADC) map of MRI are accurate definitions of the haemodynamic/metabolic changes in clinical practice reflecting the sustained electrical activity of epileptic neurons, which could help to diagnose partial NCSE [13, 14]. The MRI findings were considered TPMA. MRI tends to be a useful and easily available tool for identifying TPMA related to NCSE, which can be seen in either the ictal or postictal period of NCSE. In NCSE, the activated cortex is in an extreme electrophysiological state that exhibits increased glucose and oxygen usage, thereby causing compensatory regional hyperperfusion. Subsequently, pathophysiologic events (i.e., anaerobic glycolysis, production of lactic acid, reduction of ATP, failure of Na+/K + -ATPase and the cell membrane) lead to cytotoxic oedema and water diffusion restriction [15]. A high signal on diffusion-weighted MRI, decreased signal intensity in the ADC map, hyperintensities in T2 weighted, fluid-attenuated inversion recovery sequences and ictal hyperperfusion in MR perfusion can appear simultaneously [16]. Prolonged SE and lateralized periodic discharges are strongly associated with TPMA, and mesiotemporal structures are highly susceptible to ictal damage. Meanwhile, good spatial concordance was observed between cortical TPMA location and the EEG focus [17].

In conclusion, acute visual impairment can present as a main symptom of NCSE when the temporoparietal occipital lobes are involved, and its possible mechanism may be hemianopsia and visual neglect. At the same time, the MRI changes of TPMA could help to diagnose the disease.

## Data Availability

All data related to this case report are documented within this manuscript.

## Abbreviations

ADC: Apparent diffusion coefficient
ASL: Arterial spin labelling
EEG: Electroencephalography
MELAS: Mitochondrial encephalomyopathy with lactic acidosis and stroke-like episodes
NCSE: Nonconvulsive status epilepticus
RPLS: Reversible posterior leukoencephalopathy syndrome
TPMA: Transient peri-ictal MRI abnormalities

## References

[1] Sutter R, Semmlack S, Kaplan PW (2016). Nonconvulsive status epilepticus in adults - insights into the invisible. Nat Rev Neurol.

[2] Trinka E, Cock H, Hesdorffer D, Rossetti AO, Scheffer IE, Shinnar S, Shorvon S (2015). Lowenstein DH:a definition and classification of status epilepticus--report of the ILAE task force on classification of status Epilepticus. Epilepsia.

[3] Sutter R, Fuhr P, Grize L, Marsch S, Rüegg S (2011). Continuous video-EEG monitoring increases detection rate of nonconvulsive status epilepticus in the ICU. Epilepsia.

[4] Abboud H, Sabbagh C (2008). Acute blindness. Emerg Med J.

[5] Vilela P (2017). Acute stroke differential diagnosis: stroke mimics. Eur J Radiol.

[6] Veronelli L, Bovo S, De Giampaulis P, Passaro I, Corbo M (2016). Unilateral spatial neglect as unique clinical expression of non-convulsive status epilepticus. Cortex.

[7] Rode G, Fourtassi M, Pagliari C, Pisella L, Rossetti Y (2017). Complexity vs. unity in unilateral spatial neglect. Rev Neurol.

[8] Becker E, Karnath HO (2007). Incidence of visual extinction after left versus right hemisphere stroke. Stroke.

[9] Nagayama M, Yang S, Geocadin RG, Kaplan PW, Hoshiyama E, Shiromaru-Sugimoto A, Kawamura M. Novel clinical features of nonconvulsive status epilepticus. F1000res. 2017:6, 1690. 10.12688/f1000research.10939.1 PMC5605999 28979770

[10] Salanova V, Andermann F, Olivier A, Rasmussen T, Quesney LF (1992). Occipital lobe epilepsy: electroclinical manifestations, electrocorticography, cortical stimulation and outcome in 42 patients treated between 1930 and 1991. Surgery of occipital lobe epilepsy. Brain.

[11] O'Connor A, Costello DJ (2017). Occipital ulegyria causing epilepsy and visual impairment: an easily overlooked epilepsy syndrome. Epileptic Disorders.

[12] Leitinger M, Trinka E, Gardella E, Rohracher A, Kalss G, Qerama E, Höfler J, Hess A, Zimmermann G, Kuchukhidze G, Dobesberger J, Langthaler PB, Beniczky S (2016). Diagnostic accuracy of the Salzburg EEG criteria for non-convulsive status epilepticus: a retrospective study. Lancet Neurol.

[13] Di Bonaventura C, Bonini F, Fattouch J, Mari F, Petrucci S, Carnì M, Tinelli E, Pantano P, Bastianello S, Maraviglia B, Manfredi M, Prencipe M, Giallonardo AT (2009). Diffusion-weighted magnetic resonance imaging in patients with partial status epilepticus. Epilepsia.

[14] Shimogawa T, Morioka T, Sayama T, Haga S, Kanazawa Y, Murao K, Arakawa S, Sakata A, Iihara K (2017). The initial use of arterial spin labeling perfusion and diffusion-weighted magnetic resonance images in the diagnosis of nonconvulsive partial status epileptics. Epilepsy Res.

[15] Yogarajah M, Duncan JS (2008). Diffusion-based magnetic resonance imaging and tractography in epilepsy. Epilepsia.

[16] Mendes A, Sampaio L (2016). Brain magnetic resonance in status epilepticus: a focused review. Seizure.

[17] Giovannini Giada, Kuchukhidze Giorgi, McCoy Mark R., Meletti Stefano, Trinka Eugen (2018). Neuroimaging alterations related to status epilepticus in an adult population: Definition of MRI findings and clinical-EEG correlation. Epilepsia.

